# Real-world brain volumetry in multiple sclerosis: importance of methodological consistency and clinical relevance of gray matter atrophy

**DOI:** 10.3389/fneur.2025.1637835

**Published:** 2025-10-16

**Authors:** Joke Temmerman, Ann Vanremoortel, Maria Bjerke, Marie B. D’Hooghe, Guy Nagels, Ayla Pauwels, Diana Sima, Dirk Smeets, Jeroen Van Schependom, Mandy M. J. Wittens, Sebastiaan Engelborghs, Miguel D’haeseleer

**Affiliations:** ^1^Vrije Universiteit Brussel, Center for Neurosciences, NEUR and/or AIMS Research Groups, Brussels, Belgium; ^2^Department of Neurology, Universitair Ziekenhuis Brussel, Brussels, Belgium; ^3^Department of Biomedical Sciences, Universiteit Antwerpen, Antwerp, Belgium; ^4^Nationaal Multiple Sclerose Centrum, Melsbroek, Belgium; ^5^Department of Clinical Biology, Laboratory of Clinical Neurochemistry, Universitair Ziekenhuis Brussel, Brussels, Belgium; ^6^Division of Clinical Geriatrics, Center for Alzheimer Research, Department of Neurobiology, Care Sciences and Society, Karolinska Institutet, Stockholm, Sweden; ^7^Icometrix, Leuven, Belgium; ^8^Department of Electronics and Informatics, Vrije Universiteit Brussel, Brussels, Belgium

**Keywords:** multiple sclerosis, brain volume loss, gray matter, real-world, disability progression, post-acquisition harmonization

## Abstract

**Background:**

Brain volume loss (BVL) is a marker of neurodegeneration associated with clinical disability in multiple sclerosis (MS). However, its application in routine clinical practice is limited due to measurement errors introduced by the use of different magnetic resonance imaging (MRI) scanners across and within centers.

**Objective:**

To confirm the existence and clinical relevance of longitudinal BVL in a real-world MS cohort with scanner variability, employing a dedicated quantification pipeline combined with post-acquisition harmonization.

**Methods:**

We analyzed MRI data from 72 MS patients scanned across multiple Belgian centers over 48–60 months. Clinical disability was assessed using the Expanded Disability Status Scale, Timed 25-Foot Walk Test, 9-Hole Peg Test (9HPT), and Symbol Digit Modalities Test. Percentage volume change (PVC) in whole brain (WB), total gray matter (TGM), cortical gray matter (CGM), and deep gray matter was quantified using the icobrain ms pipeline. A similarity index was applied to account for scanner differences. Twenty-seven healthy volunteers served as controls.

**Results:**

No significant differences in annualized PVC were observed between MS patients and controls. Within the MS group, 9HPT performance correlated with TGM (*ρ* = −0.30, *p* = 0.017) and CGM (*ρ* = −0.31, *p* = 0.015) volume loss. Modified MS Functional Composite scores correlated with WB (*R* = 0.28, *p* = 0.03), TGM (*ρ* = 0.31, *p* = 0.014), and CGM (*ρ* = 0.31, *p* = 0.013) volume loss and could be independently predicted by these measures.

**Conclusion:**

Using automated brain volumetry with post-acquisition harmonization to address scanner variability, we did not detect accelerated BVL in this real-world MS cohort compared to healthy individuals. Nonetheless, GM volume loss was found to be clinically relevant in MS.

## 1 Introduction

Multiple sclerosis (MS), a chronic inflammatory demyelinating and degenerative disorder of the central nervous system (CNS), affects nearly three million people worldwide. It is the most common cause of non-traumatic neurological disability in young to middle-aged adults (1). Clinical deterioration is essentially driven by neuronal loss, which may be the consequence of (i) acute damage in newly-formed demyelinating lesions, resulting from recurrent autoimmune responses mediated by the peripheral immune system, and/or (ii) a more gradually installing neurodegeneration. The latter is believed to arise from a (non-exclusive) combination of mitochondrial dysfunction in chronically demyelinated axons, submeningeal lymphocytic clustering with damage to the underlying cortex, and pathogenic microglial activity around slowly expanding focal lesions and/or diffusely throughout the white matter (2, 3). MS is traditionally categorized into relapsing–remitting (RR), secondary progressive (SP) and primary progressive (PP) subtypes based on clinical presentation. In RR MS, tissue injury is primarily attributed to acute demyelinating lesions, whereas chronic neurodegeneration predominates in the progressive phenotypes. Recent literature endorses the stance that those phenotypes should not be seen as strictly separated entities but rather as a spectrum in which both key processes often occur together, albeit in varying proportions (3–6).

T1 contrast-enhancing and T2 hyperintense lesions are well-established magnetic resonance imaging (MRI) biomarkers of acute inflammation in MS (7). These markers are widely used in clinical practice to aid diagnosis and monitor disease activity; the latter refers to the occurrence of relapses or new focal lesions on MRI. However, their correlation with clinical outcomes is modest and often inconsistent, a phenomenon commonly named as the clinico-radiological paradox (7).

Brain volume loss (BVL) has emerged as a complementary MRI biomarkers, reflecting neurodegeneration in MS. BVL correlates with concurrent and future disability, both physical and cognitive, even in early stage of the disease (8, 9). Histological studies have demonstrated a strong association between cortical thickness measured on MRI and post-mortem brain samples, supporting the validity of BVL as a reliable indicator of actual brain atrophy (10).

BVL has increasingly been incorporated as primary or secondary endpoint in immunomodulating disease-modifying treatment (DMT) trials (11–14). A large meta-analysis evaluated over 13,000 patients across 13 pivotal studies. Brain volume changes were measured starting 6–12 months after treatment initiation to account for potential pseudo-atrophy bias, which is an apparent reduction in brain volume that can occur early after anti-inflammatory treatment due to resolution of oedema rather than neurodegeneration (15). The results showed that the effect of a therapy on BVL significantly correlates with its impact on disability outcomes, independent of its anti-inflammatory properties (16). To distinguish disease-related from age-related brain changes, a cut-off of −0.4% annual percentage volume change (PVC) has been proposed to define “pathological” BVL, offering 80% specificity and 65% sensitivity in categorizing patients (17).

Despite its research relevance, BVL has not yet been widely implemented in routine clinical practice (18). Technical factors, such as the use of different scanners, can introduce variability that often exceeds the magnitude of actual brain volume changes in uncontrolled settings (19). Only a few real-world studies in MS have assessed the value of longitudinal BVL using different scanners and primarily relied on statistical adjustments (20–22), which may limit the clinical applicability of their findings.

Our study addresses this gap by investigating the clinical relevance of longitudinal BVL over 4–5 years in a real-world MS cohort, explicitly accounting for scanner variability using a similarity index as a post-acquisition harmonization method. We employed the registration-based icobrain ms algorithm (icometrix, Leuven, Belgium), a CE-marked and FDA-cleared automated method to quantify BVL in MS (23). By combining robust real-world data with reliable volumetric quantification while also controlling for scanner variation, our study advances previous works and provides actionable insights for the potential integration of BVL into clinical monitoring.

## 2 Methods

### 2.1 Objectives and study design

**Figure 1:**
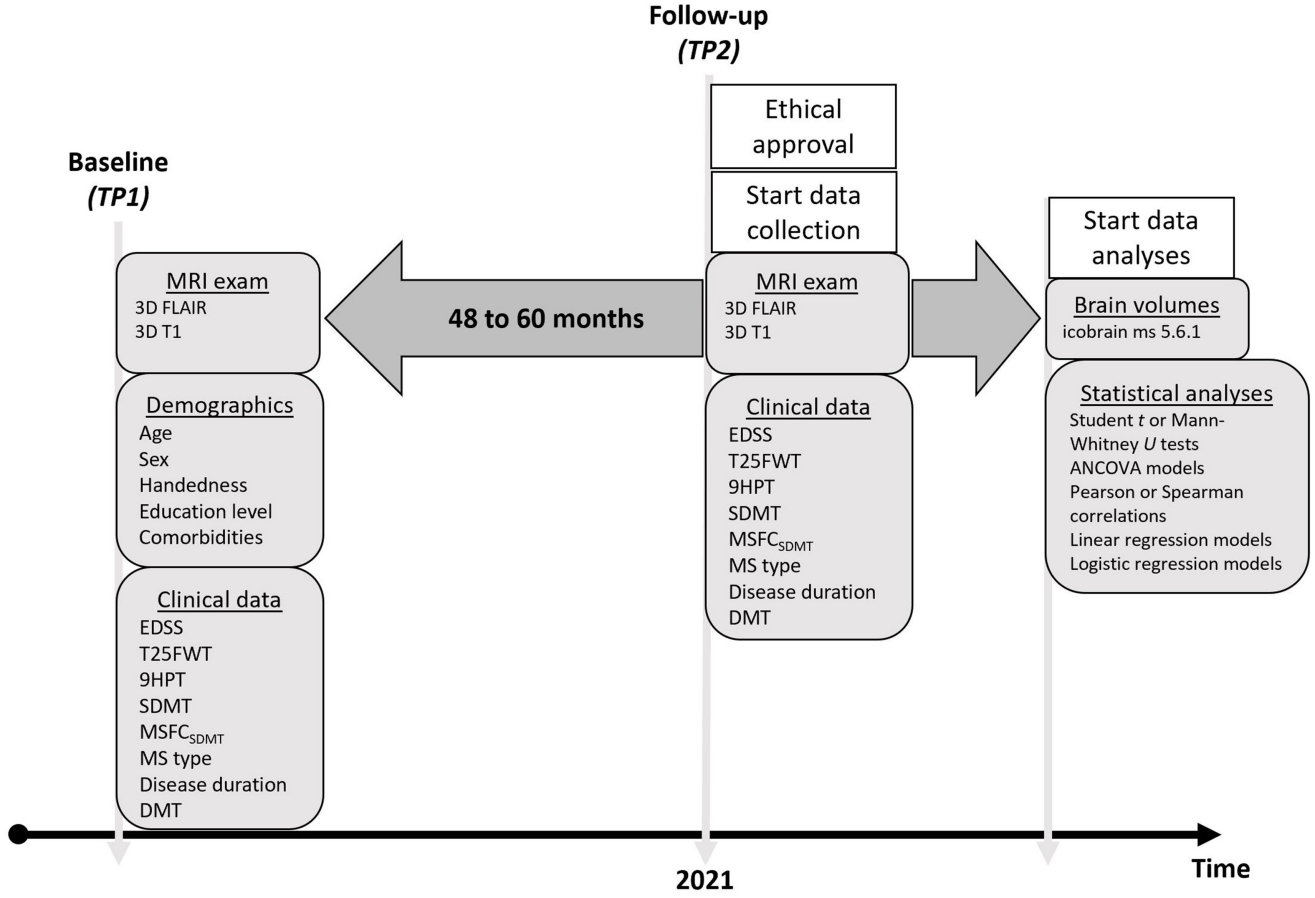
Timeline of the observational study demonstrating which variables were retrospectively collected at baseline and follow-up. TP = timepoint; EDSS = Expanded Disability Status Scale; T25FWT = Timed 25-Foot Walk Test; 9HPT = 9-Hole Peg Test; Symbol Digit Modalities Test; MSFC = Multiple Sclerosis Functional Composite; DMT = Disease Modifying Treatment; MRI = Magnetic Resonance Imaging.

This study addressed three specific research objectives. The primary objective was (1) to assess whether annualized BVL differs between patients with MS and healthy controls (HC). The secondary objectives were (2) to investigate whether BVL is associated with the evolution of clinical outcome parameters in patients with MS, and (3) to evaluate whether baseline clinical characteristics can predict “pathological” BVL (defined as > − 0.4% per year) over the observation period (17). Figure 1 provides an overview of the design of this retrospective longitudinal study. The research was conducted at the Nationaal Multiple Sclerose Centrum (NMSC) Melsbroek, a tertiary center specialized in the neurological and multidisciplinary care of patients with MS, and at the Universitair Ziekenhuis (UZ) Brussel, a university hospital, both located in Belgium. Ethical approval was granted by the Ethics Committee of the NMSC Melsbroek on June 1st 2021 (institutional authorization number: OG 033; internal reference number: EC21/06). According to the Belgian law, retrospective studies do not require participant consent. All data were de-identified prior to analysis: database and electronic health record identifiers were linked only for MRI retrieval, after which each patient was assigned a unique BRAVOLO code. All other identifiers were deleted, with a securely stored encrypted decoding file kept solely for contingency.

### 2.2 MS cohort

#### 2.2.1 MRI data

**Figure 2:**
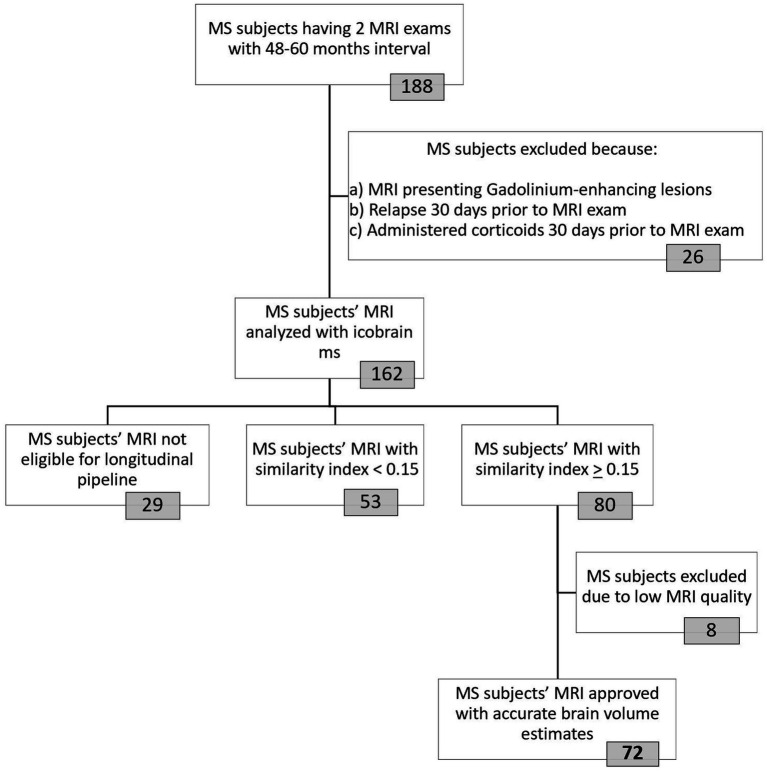
Selection procedure of final MS cohort used for data-analyses. The icobrain ms software was used to analyze MRI images from 162 included MS subjects. This fully automated registration-based method works in two steps: first, a crosssectional pipeline enables preprocessing and segmentation (gray matter, white matter, cerebrospinal fluid) of the 3D T1-weighted MRI images at each timepoint. In the second step, the longitudinal pipeline, affine registration, non-rigid registration in both directions (from TP1-TP2 and vice versa) and Jacobian integration of deformation field allows for brain volume measurement. Images from 29 MS patients were not eligible for longitudinal analyses due to invalid/corrupt input data (*n* = 13) or differences in contrast enhancement (pre-contrast versus post-contrast T1 images at the two timepoints, *n* = 16). To account for the use of different MRI machines, a similarity index with a cut-off of 0.15 was implemented. Icometrix provides detailed reports of their findings, including results from their automated quality control that tag the images as “approved,” “approved with remarks” or “rejected.” MRI images from patients with the tag “approved with remarks” were manually double-checked to determine whether they could be included. Eight MS patients were excluded because their (cross-sectional and/or longitudinal) volume estimates were inaccurate due to low tissue contrast (*n* = 6), suboptimal alignment between images (*n* = 2) and/or failed coverage of the entire brain (*n* = 1). MRI = Magnetic Resonance Imaging; MS = Multiple sclerosis; TP = Timepoint.

The selection process for our MS cohort is shown in Figure 2. We identified all subjects between 2012 and 2021 with a diagnosis of clinically definite MS, according to the McDonald 2017 criteria (24), of whom at least two MRI examinations were available in the clinical database of NMSC Melsbroek. MRI scans were obtained from routine clinical practice across various centers in Belgium. We selected the most recent MRI scan as our starting point (TP2). From there we went back in time, between 4 and 5 years (rounding allowed), to determine the baseline MRI (TP1). Automated brain volume quantification was performed using the icobrain MS software (version 5.6.1) for which the method and validation have been described earlier (23, 25). To be eligible for volumetric analysis using this pipeline, scans were required to include 3D fluid-attenuated inversion recovery (FLAIR) and T1-weighted sequences. Other MRI acquisition parameters were not pre-specified.

Patients were excluded if they had experienced a relapse or received a pulse steroid treatment within 30 days prior to either MRI examination, or if gadolinium-enhancing lesions were present on any scan. This was done to avoid the pseudo-atrophy effect, in which apparent brain volume reduction does not reflect actual neurodegeneration (15). The paired MRI scans were pseudonymized and transferred to the icobrain ms research server to calculate the longitudinal PVC for whole brain (WB), total gray matter (TGM), cortical gray matter (CGM), and deep gray matter (DGM) (23). We used the following formula to annualize the PVC values, which was designed in collaboration with researchers at icometrix:


PVC(days betweenTP1andTP2365.25)

To address scanner-related variability in MRI data, we applied the similarity index as a post-acquisition harmonization approach. The similarity index is a global quality metric summarizing technical and anatomical differences between two MRI acquisitions into one variable, based on the calculation of the normalized mutual information by linear registration of those two images, with a high value indicating a higher level of similarity (26). While a threshold of 0.20 might have been better for assessing reliability at the individual level, based on a previous scan-rescan study by Sima and colleagues (26), applying this cutoff would have excluded too many participants. Therefore, in agreement with researchers from icometrix, we adopted a threshold of 0.15 to retain a sufficient number of participants while still providing robust and reliable results at the group level. The quality of MRI images and the resulting volumes were assessed using an automated quality control (QC) system designed by icometrix to flag scans that need further visual inspection for issues (27). MRI scans flagged as “approved with remarks” were then manually reviewed for potential issues, including scan artifacts (i.e., wrapping, ringing, striping, blurring, ghosting, spiking, and susceptibility artifacts), incomplete head coverage, low tissue contrast, suboptimal image alignment and high noise levels. Manual review was performed by an experienced researcher (MMJW) to determine whether they could be included in the analysis.

Matching MRI pairs were retrieved for 162 patients with MS, which were subsequently processed by the icobrain ms software. Only 44% (*n* = 72) of the initial cohort was found eligible for final inclusion based on the similarity index with cut-off 0.15 and the QC systems. In this study group, both 1.5 T and 3 T MRI were used, obtained from four different manufacturers: Philips, Siemens, GE and Olea Medical (Supplementary Table 1). The majority of participants had no change in field strength (76%) or manufacturer (71%) between TP1 and TP2.

#### 2.2.2 Clinical data

Demographic patient data at the time of TP1 were collected from the medical records and included age, sex, disease onset, disease duration, disease-modifying treatments (DMT; first-line: interferon beta-1A, interferon beta-1B, dimethyl fumarate, glatiramer acetate, and teriflunomide; second-line: natalizumab, fingolimod, and ocrelizumab), MS phenotype (secondary and primary progressive MS was combined as progressive MS, PMS), education level and the presence of cardiovascular risk factors/comorbidities (smoking, diabetes mellitus, arterial hypertension, hypercholesterolemia, and obesity; the latter defined as a body mass index of 30 or more). Clinical disability was assessed using multiple outcome measures: Expanded Disability Status Scale (EDSS) for general disability (28), Timed 25-Feet Walk Test (T25FWT) for walking function (29), dominant hand 9-Hole Peg Test (9HPT) for dexterity (30), and Symbol Digit Modalities Test (SDMT) scores for cognition (31). These variables were extracted when evaluated around the same time as TP1 and TP2, not more than 6 months before or after each MRI scan. We used a composite measure at baseline and follow-up based on the Z-scores of the T25FWT, 9HPT, and SDMT, to generate a modified Multiple Sclerosis Functional Composite (MSFC_SDMT_) Score (32). A Z-score indicates how far a patient’s score deviates from the mean value of a reference population. We derived mean values and standard deviations from the National MS Society Task Force database, which represents a broad spectrum of MS patients, to calculate these Z-scores (27). Additionally, we calculated the change in clinical scores by determining the difference between follow-up and baseline measurements, resulting in δEDSS, δT25FWT, δ9HPT, δSDMT, and δMSFC_SDMT_ values. For clinical interpretation, a higher score on the EDSS, T25FWT, and 9HPT reflects a higher level of disability, while a higher score on the SDMT and MSFC_SDMT_ reflects a lower level of disability. Thus, positive changes (meaning higher scores at TP2 compared to TP1) indicate worsening disability according to the EDSS, T25FWT, and 9HPT, while suggesting improvement according to the SDMT and MSFC_SDMT_. Of the 72 MS patients in our study group, 62 had complete clinical data available.

### 2.3 HC cohort

We used a historic HC cohort that was established via a different study, which included appropriate institutional board approval and written informed consent, from the same research team (33). In brief, volunteers had undergone two MRI exams, with an interval of at least 1 year, between 2015 and 2020, in a controlled study environment set up at UZ Brussel. Imaging was done with a 3 T scanner (Achieva, Philips Medical Systems) that included 3D T2-weighted FLAIR and T1-weighted sequence with the following parameters: 310 sagittal slices, TR = 4.939 ms, FOV = 230 × 230 mm^2^, voxel resolution 0.53 × 0.53 × 0.5 mm^3^. Demographic variables were re-used, but BVL was *de novo* analyzed using the same version of the processing pipeline from icometrix as the one employed in the MS cohort.

### 2.4 Statistical analyses

The three study objectives are outlined above (section 2.1). For the primary endpoint of this study (1), annualized PVC for WB, TGM, CGM and DGM, were compared between patients with MS and HC. Following a Shapiro–Wilk test for normality, group differences were recorded with unpaired Student t or Mann–Whitney U tests, where appropriate. ANCOVA models were used to check for influences of potential confounders. All analyses addressing the primary objective were performed in the complete MS study group (*n* = 72). For secondary objectives requiring complete clinical data, analyses were restricted to a secondary analysis cohort (*n* = 62). Within this secondary analysis cohort, we first stratified patients with and without “disability worsening,” based on the established cut-offs for deterioration in EDSS, T25FWT, 9HPT and SDMT scores, respectively, as defined in Table 1 (34). We then investigated differences in PVC between patients with MS who showed “disability worsening” and those that did not. All subsequent secondary analyses were conducted in the entire secondary analysis cohort unless otherwise specified. For secondary objective (2), the relationship between PVC and the evolution of clinical outcome parameters was analyzed using Pearson or Spearman’s rank correlation coefficients and linear regression models. The linear regression models were constructed using the change in clinical scores over time as outcome variables and the respective PVC measures as predictors. Scatter plots illustrating the normality of the residuals and homoskedasticity were visually checked and multicollinearity was avoided. With stepwise forward inclusion potential confounders (age, sex, cardiovascular risk factors/comorbidities, education, DMT, MS phenotype, and disease duration) were added in significant models to evaluate their influence on the relation between clinical and MRI parameters. Categorical confounders were decoded as followed: sex (female; male), presence of cardiovascular health comorbidities (none; 1; ≥ 2), education (≥ 12 years starting from elementary school = “higher”; < 12 years starting from elementary school = “lower”), DMT (none; first-line; second-line) and MS phenotype (RR; PMS). Likewise, we performed logistic regression for (secondary) objective (3), to assess whether baseline demographics or measures of clinical status (i.e., EDSS, T25FWT, 9HPT, SDMT, MSFC_SDMT_) could be predictive for “pathological” WB volume loss (see definition above).

**Table 1:** Cut-offs used to categorize MS patients as “disability worsening.”

Clinical score	Criteria for disability worsening
EDSS	Increase of 1.5 points if baseline score is 0
	Increase of 1.0 point if baseline score is between 1.0 and 5.5
	Increase of 0.5 point if baseline score is higher than 5.5
T25FWT/9HPT	Significant change of ≥ 20%
SDMT	Reduction of ≥ 4 points or a 10% worsening

9HPT = 9-Hole Peg Test; EDSS = Expanded Disability Status Scale; SDMT = Symbol Digit Modalities Test; T25FWT = Timed 25-Foot Walk Test. Based on Meca-Lallana et al. (34).

Statistical analyses were performed in R (version 4.0.5; Auckland, New Zealand). All reported *p* values are two-tailed with statistical significance set at 0.05. This study was conducted according to “The Strengthening the Reporting of Observational Studies in Epidemiology” (STROBE) statement for reporting observational cohort studies (Supplementary Table 2) (35).

## 3 Results

### 3.1 BVL in patients with MS versus HC

Following the selection process, our MS group consisted of 72 patients (59 RR MS and 13 PMS). Seventy-two percent was on DMT: interferon beta-1A (*n* = 4), interferon beta-1B (*n* = 10), dimethyl fumarate (*n* = 9), glatiramer acetate (*n* = 8), teriflunomide (*n* = 5), natalizumab (*n* = 10), fingolimod (*n* = 5), and ocrelizumab (*n* = 1). We observed less BVL for TGM, CGM and DGM in patients on second-line agents as compared to those on first-line. However, these differences were not statistically significant (Table 2). The HC cohort consisted of 27 subjects with a median similarity index of 0.29%. We found significant differences in median similarity index and interscan interval between both groups, but not in age, sex or annualized PVC of WB, TGM, CGM or DGM (Table 3).

**Table 2:** Differences in BVL between different DMT modalities.

Brain volume measures	No DMT (*n* = 20)	First-line (*n* = 36)	Second-line (*n* = 16)	*p*-value
Annualized PVC WB	−0.18 ± 0.43	−0.17 ± 0.26	−0.18 ± 0.26	0.360
Annualized PVC TGM	−0.33 ± 0.37	−0.27 ± 0.30	−0.19 ± 0.31	0.407
Annualized PVC CGM	−0.32 ± 0.38	−0.27 ± 0.31	−0.10 ± 0.30	0.473
Annualized PVC DGM	−0.47 [0.82]	−0.50 [0.68]	−0.30 [0.97]	0.701

Data presented in mean ± SD or median [range]. CGM = Cortical Gray Matter; DGM = Deep Gray Matter; DMT = Disease-modifying treatment; PVC = Percentage Volume Change; SD = Standard Deviation; TGM = Total Gray Matter; WB = Whole Brain. First-line DMT includes dimethyl fumarate (*n* = 9), glatiramer acetate (*n* = 8), interferon beta-1A (*n* = 4), interferon beta-1B (*n* = 10) and teriflunomide (*n* = 5). Second-line treatment includes fingolimod (*n* = 5), natalizumab (*n* = 10) and ocrelizumab (*n* = 1).

**Table 3:** Demographics of MS and HC cohorts.

Characteristics	MS	HC	MS vs HC *p*-value
Number of subjects	72	27	NA
Age (years)	45 ± 9	49 ± 13	0.180
Sex (F/M)	57/15 (79.2%)	16/11 (59.3%)	0.071
MS phenotype (RR/PMS)	59 / 13	NA	NA
DMT (None/First-line/Second-line)	20/36/16	NA	NA
Interscan interval (months)	53 [43–62]	32 [21–49]	**< 0.001**
Similarity-index	0.21 [0.15–0.34]	0.29 [0.24–0.44]	**< 0.001**
Annualized PVC WB	−0.17 ± 0.31	−0.29 ± 0.27	0.055
Annualized PVC TGM	−0.27 ± 0.32	−0.35 ± 0.29	0.238
Annualized PVC CGM	−0.27 ± 0.33	−0.34 ± 0.29	0.271
Annualized PVC DGM	−0.45 [−2.30–1.32]	−0.55 [−2.22–0.11]	0.430

Continuous data presented in mean ± SD or median [range], categorical data. CGM = Cortical Gray Matter; DGM = Deep Gray Matter; DMT = Disease-modifying treatment; F = Female; HC = Healthy controls; M = Male; MS = Multiple Sclerosis; NA = Not Applicable; PMS = Progressive Multiple Sclerosis; PVC = Percentage Volume Change; RR = Relapsing–Remitting; SD = Standard Deviation; TGM = Total Gray Matter; WB = Whole Brain. First-line DMT includes dimethyl fumarate (*n* = 9), glatiramer acetate (*n* = 8), interferon beta-1A (*n* = 4), interferon beta-1B (*n* = 10) and teriflunomide (*n* = 5). Second-line treatment includes fingolimod (*n* = 5), natalizumab (*n* = 10) and ocrelizumab (*n* = 1). Significant *p*-values are shown in bold.

Several additional *post hoc* analyses were performed to see whether potential confounders influenced the between group comparisons. We first evaluated the effect of DMT by comparing BVL between HC and MS subjects not receiving DMT (28%), but results were similar to those observed for the entire MS cohort (Supplementary Table 3). Although the mean age of patients with MS and HC cohorts was comparable (Table 3), visual inspection of the boxplots did suggest an unequal distribution. When categorizing both cohorts by age, we observed that the HC had the highest fraction of individuals over 55 years of age, whereas this age category was a minority in the MS cohort (Supplementary Figure 1). This figure also illustrates accelerated BVL with increasing age in both groups. Additionally, the similarity index was significantly higher in the HC (Table 3), who had no scanner changes, as compared to the MS cohort, where 29% experienced a manufacturer change and 21% experienced a field strength change. To account for these confounders, an ANCOVA was performed to analyze differences in annualized PVC of WB between MS and HC cohorts, controlling for age and similarity index. Age emerged as a significant predictor of BVL (*p* = 0.002), and the similarity index had a borderline significant effect (*p* = 0.053). These findings suggest that our rather unexpected observation of similar BVL in patients with MS and HC may have been due to differences in age distribution and, to a lesser extent, in similarity index.

### 3.2 Brain volume change and clinical disability progression in patients with MS

**Figure 3:**
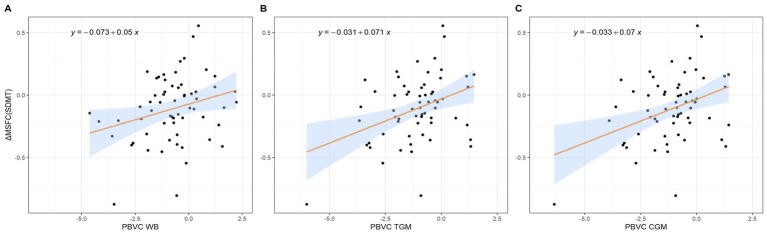
Scatterplots representing the relation between the change in MSFC_SDMT_ and **(A)** percentage whole brain volume change, **(B)** percentage total gray matter volume change and **(C)** percentage cortical gray matter volume change. CGM = Cortical Gray Matter; MSFC_SDMT_ = Multiple Sclerosis Functional Composite using SDMT; PVC = Percentage Volume Change; TGM = Total Gray Matter; WB = Whole Brain.

We found a significant decrease in TGM and CGM PVC over time in patients demonstrating disability worsening based on the 9HPT test, as compared with those that did not (Table 4). For the complete secondary analysis cohort, we found a negative correlation between the δ9HPT scores and PVC for TGM (*ρ* = −0.30, *p* = 0.017) and CGM (*ρ* = −0.31, *p* = 0.015). There was a positive correlation between δMSFC_SDMT_ scores and PVC WB (*R* = 0.28, *p* = 0.03), TGM (*ρ* = 0.31, *p* = 0.014) and CGM (*ρ* = 0.31, *p* = 0.013). Regression modeling revealed that clinical worsening according to the δMSFC_SDMT_ could be predicted by changes in WB (*β* = 0.05, SE = 0.02, *p* = 0.03), TGM (*β* = 0.07, SE = 0.02, *p* = 0.002), and CGM (*β* = 0.07, SE = 0.02, *p* = 0.003) volumes without being influenced by potential confounders (age, sex, education level, presence of cardiovascular comorbidities, disease duration, DMT or MS phenotype) (Table 5; Figure 3; Supplementary Table 4).

**Table 4:** Comparison of brain volume measures between “disability worsening” and “stable disability” MS patients according to the EDSS, T25FWT, 9HPT and SDMT change over time.

Worsening based on EDSS change	Brain volume measures	Disability worsening (*n* = 24)	Stable disability (*n* = 38)	Worsening vs Stable *p*-value
	PVC WB	−0.85 ± 1.75	−0.68 ± 1.24	0.635
	PVC TGM	−1.67 [−3.67–1.32]	−0.86 [−6.02–1.46]	0.053
	PVC CGM	−1.59 [−3.91–1.44]	−0.82 [−6.37–1.43]	0.063
	PVC DGM	−2.44 ± 2.67	−1.40 ± 2.30	0.123

Worsening based on T25FWT change	Brain volume measures	Disability worsening (*n* = 36)	Stable disability (*n* = 26)	Worsening vs Stable *p*-value
	PVC WB	−0.61 ± 1.63	−0.92 ± 1.15	0.386
	PVC TGM	−0.86 [−6.02–1.32]	−1.01 [−3.60–1.46]	0.368
	PVC CGM	−0.82 [−6.37–1.44]	−1.01 [−3.61–1.43]	0.492
	PVC DGM	−1.71 ± 2.65	−1.92 ± 2.28	0.737

Worsening based on 9HPT change	Brain volume measures	Disability worsening (*n* = 40)	Stable disability (*n* = 22)	Worsening vs Stable *p*-value
	PVC WB	−0.89 ± 1.32	−0.47 ± 1.64	0.312
	PVC TGM	−1.10 [−6.02–1.46]	−0.37 [−2.97–1.32]	0.005*
	PVC CGM	−1.07 [−6.37–1.44]	−0.30 [−2.83–1.31]	0.003*
	PVC DGM	−1.75 [−6.63–1.59]	−1.48 [−6.82–5.64]	0.752

Worsening based on SDMT change	Brain volume measures	Disability worsening (*n* = 19)	Stable disability (*n* = 43)	Worsening vs Stable *p*-value
	PVC WB	−0.97 ± 1.46	−0.64 ± 1.44	0.417
	PVC TGM	−1.33 [−6.02–1.32]	−0.76 [−3.67–1.46]	0.132
	PVC CGM	−1.30 [−6.37–1.31]	−0.78 [−3.91–1.44]	0.140
	PVC DGM	−1.82 ± 2.98	−1.80 ± 2.27	0.980

Data presented in mean ± SD or median [range]. 9HPT = 9-Hole Peg Test; CGM = Cortical Gray Matter; DGM = Deep Gray Matter; EDSS = Expanded Disability Status Scale; MS = Multiple Sclerosis; PVC = Percentage Volume Change; SD = Standard Deviation; SDMT = Symbol Digit Modalities Test; T25FWT = Timed 25-Foot Walk Test; TGM = Total Gray Matter; WB = Whole Brain. *p < 0.01. Significant *p*-values are shown in bold.

**Table 5:** Linear regression models with clinical scores as dependent variables and MRI measures as independent variables.

Brain volume measures	ΔEDSS	ΔT25FWT	Δ9HPT	ΔSDMT	ΔMSFC_SDMT_
PVC WB	−0.03 ± 0.10	−0.05 ± 0.21	−0.04 ± 0.44	0.68 ± 0.70	0.05 ± 0.02*
PVC TGM	−0.07 ± 0.10	−0.04 ± 0.22	−0.49 ± 0.45	1.25 ± 0.71	0.07 ± 0.02 **
PVC CGM	−0.06 ± 0.10	−0.04 ± 0.21	−0.48 ± 0.43	1.23 ± 0.69	0.07 ± 0.02**
PVC DGM	−0.003 ± 0.05	−0.01 ± 0.12	0.06 ± 0.26	0.15 ± 0.41	0.001 ± 0.01

Data is presented as estimates of regression coefficient (*β*) ± SE. 9HPT = 9-Hole Peg Test; CGM = Cortical Gray Matter; DGM = Deep Gray Matter; EDSS = Expanded Disability Status Scale; MSFC_SDMT_ = Multiple Sclerosis Functional Composite Score using SDMT; PVC = Percentage Volume Change; SDMT = Symbol Digit Modalities Test; SE = Standard Error; T25FWT = Timed 25-Foot Walk Test; TGM = Total Gray Matter; WB = Whole Brain. *p < 0.05; **p < 0.01. Significant *p*-values are shown in bold.

### 3.3 Baseline clinical variables predictive of pathological brain volume change

Annualized WB PVC was dichotomized, with “pathological” BVL below −0.4%. Twelve patients with MS (19%) demonstrated “pathological” annualized WB volume loss, whereas the remaining 50 patients (81%) had “physiological” WB volume loss. There were no significant differences observed between these two groups (Table 6) and none of the baseline characteristics could significantly predict the probability of having “pathological” BVL (Table 7).

**Table 6:** Demographics of MS cohorts used for logistic regression analyses, using a cut-off of −0.4% annualized whole brain volume loss.

Characteristics	Physiological BVL (≥ − 0.4%/year)	Pathological BVL (< −0.4%/year)
Number of subjects	50	12
Age (years)	44 ± 9	49 ± 12
Sex (F/M)	40/10	10/2
CVD (None/1/>2)	27/16/7	5/5/2
MS phenotype (RR/PMS)	44/6	9/3
DMT (None/First-line/Second-line)	11/27/12	5/5/2
DMT per agent		
None	11	5
Dimethyl fumarate	7	2
Glatiramer acetate	6	1
Interferon beta-1A	4	0
Interferon beta-1B	6	2
Teriflunomide	4	0
Fingolimod	4	0
Natalizumab	7	2
Ocrelizumab	1	0
Education level (Lower/Higher)	24/26	8/4
Interscan interval (months)	53 ± 5	54 ± 5
Disease duration (months)	150 [36–396]	192 [24–408]
Baseline EDSS	3.0 [1.0–6.5]	4.0 [1.5–6.5]
Baseline T25FWT (seconds)	5.3 [3.2–16.5]	6.0 [4.9–15.0]
Baseline 9HPT Dominant (seconds)	20.6 [14.0–38.3]	20.9 [16.2–37.1]
Baseline SDMT	52.2 ± 13.4	48.9 ± 14.6
Baseline MSFC_SDMT_	0.4 ± 0.7	0.2 ± 0.7

Data presented in mean ± SD or median [range]. 9HPT = 9-Hole Peg Test; BVL = Brain volume loss; CVD = Cardiovascular comorbidities; DMT = Disease-modifying Treatment; EDSS = Expanded Disability Status Scale; F = Female; M = Male; MSFC_SDMT_ = Multiple Sclerosis Functional Composite Score using SDMT; PMS = Progressive Multiple Sclerosis; RR = Relapsing–Remitting; SD = Standard Deviation; SDMT = Symbol Digit Modalities Test; T25FWT = Timed 25-Foot Walk Test.

**Table 7:** Logistic regression models for annualized whole brain volume loss with a cut-off of −0.40% per year as dependent variables and baseline clinical and demographic measures as independent variables.

Baseline measures	Pathological BVL (< −0.4% per year)	
EDSS	0.59 ± 0.43	
T25FWT	−0.21 ± 0.24	
9HPT	−0.15 ± 0.23	
SDMT	0.09 ± 0.14	
MSFC	−2.38 ± 4.03	
Age	0.03 ± 0.05	
Sex (Male)	−0.81 ± 1.10	
Disease duration	−0.001 ± 0.06	
MS phenotype (RR MS)	0.20 ± 1.15	
Education level (Higher)	−1.00 ± 0.88	
Comorbidities (One/Two or more)	0.26 ± 0.97	0.17 ± 1.13
DMT (First-line/Second-line)	−0.65 ± 0.92	−1.77 ± 1.32

Data is presented as estimates of regression coefficient (β) ± SE. 9HPT = 9-Hole Peg Test; BVL = Brain volume loss; DMT = Disease-modifying Treatment; EDSS = Expanded Disability Status Scale; F = Female; M = Male; MSFC_SDMT_ = Multiple Sclerosis Functional Composite Score using SDMT; RR = Relapsing–Remitting; SDMT = Symbol Digit Modalities Test; SE = Standard Error; T25FWT = Timed 25-Foot Walk Test. First-line DMT includes dimethyl fumarate (*n* = 9), glatiramer acetate (*n* = 8), interferon beta-1A (*n* = 4), interferon beta-1B (*n* = 10) and teriflunomide (*n* = 5). Second-line treatment includes fingolimod (*n* = 5), natalizumab (*n* = 10) and ocrelizumab (*n* = 1).

The number of patients showed an unequal distribution amongst both groups, prompting our hypothesis whether another cut-off could have been more informative. When using a cut-off based on the observed mean WB PVC in the MS cohort, (i.e., below −0.16% PVC), there were 28 individuals with “pathological” and 34 with “physiological” WB volume loss. A significant difference in baseline T25FWT score was observed between these two groups (Supplementary Table 5, *p* = 0.034). However, regression analyses once again failed to detect baseline variables that independently predict the probability of reaching “pathological” BVL (Supplementary Table 6).

## 4 Discussion

BVL has recently gained attention as an MRI-derived proxy for neurodegeneration in patients with MS. The implementation of brain volumetry into routine clinical practice, though, has been hindered, mainly due to the risk of measurements becoming less precise once they are conducted outside strictly standardized research settings. In this longitudinal study, we aimed to validate the clinical relevance of BVL in a real-world cohort of patients with MS while explicitly taking into scanner differences using a similarity index as post-acquisition harmonization approach. We did not observe significant differences in annualized BVL between MS and HC essentially failing the primary objective of this study. However, worsening functional ability in patients with MS, as measured by 9HPT scores, was linked to increased atrophy in both TGM and CGM. Additionally, clinical decline on the composite MSFC_SDMT_ outcome measure was associated with WB, TGM, and CGM volume loss. We could not predict “pathological” BVL based on baseline demographics or clinical status.

It has been widely accepted that MS patients present with accelerated BVL compared to healthy individuals (9, 20, 21). The majority of studies on BVL, both in standardized and real-world settings, use the structural image evaluation with normalization of atrophy, or SIENA, method for PVC quantification, whereas the effect of change in MRI scanner strength (1.5 or 3.0 T) over time is usually taken into account with mixed-effect regression models (20–22). We have used the icobrain ms software to quantify BVL and applied a similarity index as post-acquisition harmonization method to consider scanner switches. We found PVC rates below the typical values of brain atrophy in MS (36), with only 15 of our 72 participants (21%) actually demonstrating annualized WB volume loss below 0.4%. However, this discrepancy cannot be due to the use of another tool for PVC quantification, as consistency between both methods for real-world BVL analysis has already been demonstrated (37). The negative outcome of our first objective (primary endpoint) does not seem to be influenced by a treatment effect, but may have been due to the unequal distribution of age between the MS and HC cohorts. Recent research suggest that age-related BVL accelerates significantly around 60 years of age (38). As shown in Supplementary Figure 1, the greatest mean BVL was observed in the oldest age category (55–70 years) for both HC and MS cohorts, where there is an important imbalance between the number of people with MS and HC. This imbalance may contribute to the apparent lack of difference in brain atrophy between the groups at the overall group level. Limited statistical power may have also contributed to our negative finding to some extent. Our sample sizes were sufficient to detect medium-to-large effects, but smaller differences in BVL could have gone undetected, increasing the risk of a type II error. Finally, survivor bias may have played a role: patients with more aggressive disease courses may have been less likely to remain in long-term follow-up, resulting in an overrepresentation of more stable individuals in the MS cohort and attenuating observed differences compared to HC. Nonetheless, some other studies have also reported low brain atrophy rates in MS (39, 40), further supporting the reliability of our BVL measures. Despite having a cohort with less global BVL than expected, these patients with MS still exhibited signs of disability worsening, which we were able to connect to GM volume loss.

MS was classically considered a disease of white matter (WM), but the intensity and relevance of GM involvement has become increasingly evident over the past two decades. GM volume loss, particularly in the deep nuclei, can occur early in the disease course and independent of focal WM lesions (41, 42). Moreover, several longitudinal studies have demonstrated that the volumetric decline of GM structures is not only more pronounced as compared to WM, but also more strongly associated with clinical outcomes (43, 44). Interestingly, the increased rate of WB volume loss observed in MS patients with progression independent of relapse activity, as compared to those that remained stable, could mainly be attributed to changes in the cerebral cortex (45). Our result are in line with previous findings, reporting significant associations between (C)GM atrophy and disease worsening, according to the 9HPT score and MSFC_SDMT_ (46, 47). BVL in GM can manifest according to different spatial patterns, which is relevant to specific clinical manifestations and even phenotypes (48–50). Regional GM atrophy may more accurately reflect the status of certain individual clinical measures than global estimates. For example, thalamic atrophy seems to be associated with cognitive impairment reflected by the SDMT in patients with MS, while overall GM atrophy does not (51). While we did not find statistically significant DMT effects on BVL, possibly due to a lack of statistical power, others have recently reported an increase of thalamic volume with natalizumab, suggesting a potential neuroprotective role.^52^ So, even though we found an association between (C)GM atrophy and overall disease progression, more locally defined anatomic regions, such as the thalamus, may even be more informative about certain clinical as well as therapeutic aspects of MS. Unfortunately, parcellation of the CGM and segmentation of the DGM is not part of the default icobrain ms pipeline (and thus not validated for use in data from clinical routine) but may still be an interesting objective for future studies. Next to the importance of spatial patterns in brain atrophy, an important temporal factor might be at play when considering the intricate nature of the relationship between WM and GM pathology. A recent systematic review concluded that global GM atrophy appears to be secondary to focal WM lesions in early stages of the disease, and only later in the disease course will adopt a more independent character due to other neurodegenerative processes that are still not fully understood (52). This may correspond to the gradual change in inflammatory pathways throughout the disease course, with a predominant role of invading T lymphocytes in the formation of WM lesions in relapsing MS (1), and meningeal inflammation, amongst others, likely acting as a driving force for cortical atrophy in the progressive phase, which seemingly develops according to a clear gradient of neuronal loss that turns inwards from the boundary between GM and cerebrospinal fluid (53, 54). Such shift may even happen very early in the disease, as CIS patients that progress toward RR MS already show a 3.4 fold increase in GM atrophy, but no change in WM when compared to HC (46).

We acknowledge several limitations in the present study. First, despite our efforts to minimize the potential confounding impact of acute inflammatory activity or edema on BVL quantification, a pseudo-atrophy effect may not be limited to the presence of active lesions only. Recent literature has shown (i) that up to 25% of acute clinical events identified as relapse do not appear to be associated with lesional changes on MRI (55), (ii) contrast-enhancement within acute focal lesions typically lasts for approximately 4 weeks, whereas the pseudo-atrophy effect seems to reach a plateau only after 16 to 20 weeks (56, 57), (iii) inflammation in MS is not restricted to focal lesions and may occur diffusely throughout the normal-appearing white matter as well (58), and (iv) the exact pathophysiological mechanism behind pseudoatrophy is still not fully understood and may involve other processes (besides accelerated water loss/fluid shifts), such as changes in glial cells (15, 56). Second, during our selection process, we experienced important loss of participants that drastically reduced the number of patients in our final sample. The subgroup with pathological BVL was particularly small (*n* = 12), which may have further limited our statistical power for the final objective. Previous studies have reported drop-out rates ranging from 37 to 82% (20–22, 59), and notably, one of these indicated a higher failure rate when focusing exclusively on 3D (82%) versus 2D (56%) imaging (59). Our drop-out rate of 56% aligns with the existing literature and we included 3D acquisition only. Notably, data loss in our study more frequently stemmed from applying the similarity index with a cut-off of 0.15 (33%), than from failing to meet key technical standards (16%). Even though we decided to continue with a lower similarity index cut-off (0.15 rather than 0.20) to retain as much patients as possible, we still experienced significant data loss due to this measure. Third, the similarity index does not account for various biological factors that can influence longitudinal volume changes, such as timing of scans, hydration state, lifestyle factors, and comorbidities. Nonetheless, a recent study suggests that technical causes contribute more significantly to variations in brain volume measures than physiological factors (60), reinforcing the value of using a similarity index as a post-acquisition harmonization approach in our analysis. Fourth, we used retrospective data for the MS cohort and relied on clinical data from two timepoints only (baseline and follow-up), limiting our ability to track confirmed disability worsening. On the other hand, we did employ a range of disability measures. This comprehensive approach allows us to capture various dimensions of disability, which is crucial in a heterogeneous condition like MS. Fifth, we used the SDMT for calculating MSFC scores, whereas this metric normally involves the Paced Auditory Serial Addition Test (PASAT) (61). This does not allow us to accurately compare our findings with others from the field. Still, both scores are representatives of cognition (particularly information processing speed) and using the SDMT score instead of the PASAT may even be an improvement due to its slightly better predictive validity, particularly when considering longitudinal data (62–64). Finally, we have only included the total EDSS score without its individual functional systems (i.e., the visual, brainstem, pyramidal, cerebellar, sensory, bowel and bladder, and cerebral functions) (28). In this real-world study, collecting such detailed information during routine medical visits is often challenging due to time constraints. To overcome this limitation, we have tried to take a multifaceted approach, by incorporating various measures of disability in MS.

## 5 Conclusion

Using the automated icobrain ms algorithm with similarity index-based post-acquisition harmonization to address scanner variability, we did not detect accelerated BVL in this real-world MS cohort compared to healthy individuals. Nonetheless, GM volume loss remains clinically relevant in MS, as it was associated with disability worsening according to the 9HPT and the MSFC_SDMT_. To enable routine clinical use of BVL measurements, future research should prioritize robust post-acquisition correction methods that explicitly account for scanner differences. In the meantime, a practical recommendation for clinical settings is to perform follow-up scans on the same scanner whenever feasible, to enhance the reliability of longitudinal BVL assessments.

## Data Availability

Anonymized data supporting the findings of our study will be shared upon reasonable request from a qualified investigator and after approval by the ethics committees of NMSC Melsbroek and UZ Brussel.
