# Supplementary material for: Real-world brain volumetry in multiple sclerosis: importance of methodological consistency and clinical relevance of gray matter atrophy
Source: Front Neurol. 2025 Oct 16;16:1637835. doi: 10.3389/fneur.2025.1637835 (PMC12571658; doi:10.3389/fneur.2025.1637835)
Supplement: Supplementary file 1 [file Table_1.DOCX]

Supplementary Material

# Supplementary Figure


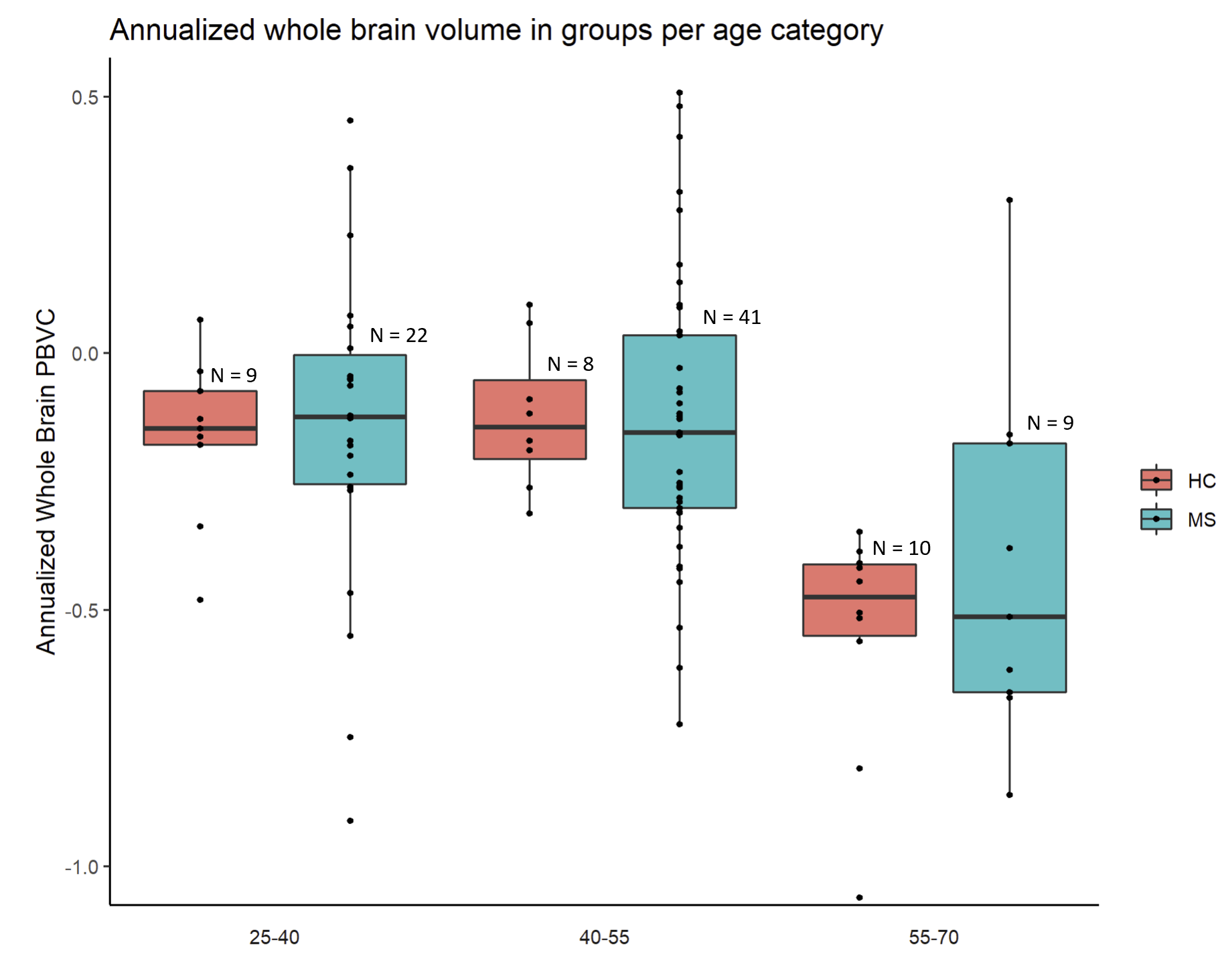


**Supplementary Figure 1.** annualized whole brain volume according to different age categories in MS and HC groups, demonstrating a higher fraction of people aged >55 in HC than MS.

# Supplementary Tables

**Supplementary Table 1.** Detailed overview of MRI equipment used for each group. Continuous data presented in mean + SD or median [range], categorical data presented as count (percentage).

|  |  | **MS (n = 72)** | **HC (n = 27)** | ***MS vs HC p-value*** |
| --- | --- | --- | --- | --- |
| **Number of subjects according to field strength changes over time** |  |  |  |  |
|  | 1.5T -> 1.5T | 19 (26%) | 0 | NA |
|  | 1.5T -> 3.0T | 7 (10%) | 0 | NA |
|  | 3.0T -> 3.0T | 36 (50%) | 27 (100%) | NA |
|  | 3.0T -> 1.5T | 10 (14) | 0 | NA |
| **Number of subjects according to manufacturer changes over time** |  |  |  |  |
|  | Philips -> Philips | 23 (32%) | 27 (100%) | NA |
|  | Siemens -> Siemens | 21 (29%) | 0 | NA |
|  | GE -> GE | 6 (9%) | 0 | NA |
|  | Olea Medical -> Olea Medical | 1 (1%) | 0 | NA |
|  | Different manufacturers | 21 (29%) | 0 | NA |
| **Similarity index** |  |  |  |  |
|  | 1.5T -> 1.5T | 0.21 [0.07] | NA | NA |
|  | 1.5T -> 3.0T | 0.18 [0.04] | NA | NA |
|  | 3.0T -> 3.0T | 0.21 [0.07] | 0.29 [0.07] | < 0.001 |
|  | 3.0T -> 1.5T | 0.17 [0.02] | NA | NA |
|  | Philips -> Philips | 0.23 [0.06] | 0.29 [0.07] | < 0.001 |
|  | Siemens -> Siemens | 0.20 [0.05] | NA | NA |
|  | GE -> GE | 0.25 [0.01] | NA | NA |
|  | Olea Medical -> Olea Medical | 0.19 [0] | NA | NA |
|  | Different manufacturers | 0.17 [0.03] | NA | NA |
| **Annualized PVC WB** |  |  |  |  |
|  | 1.5T -> 1.5T | -0.21 + 0.23 | NA | NA |
|  | 1.5T -> 3.0T | -0.37 + 0.42 | NA | NA |
|  | 3.0T -> 3.0T | -0.15 + 0.32 | -0.29 + 0.27 | 0.05402 |
|  | 3.0T -> 1.5T | -0.04 + 0.32 | NA | NA |
|  | Philips -> Philips | -0.15 + 0.35 | -0.29 + 0.27 | 0.111 |
|  | Siemens -> Siemens | -0.16 + 0.22 | NA | NA |
|  | GE -> GE | -0.13 + 0.29 | NA | NA |
|  | Olea Medical -> Olea Medical | -0.24 + NA | NA | NA |
|  | Different manufacturers | -0.22 + 0.37 | NA | NA |

HC = Healthy controls; MRI = Magnetic Resonance Imaging; MS = Multiple Sclerosis; PVC = Percentage Volume Change; SD = Standard Deviation; WB = Whole Brain.

**Supplementary Table 2:** Reporting checklist for cohort study. Based on the STROBE cohort guidelines.

|  |  | Reporting Item | Page Number |
| --- | --- | --- | --- |
| **Title and abstract** |  |  |  |
| Title | [#1a](https://www.goodreports.org/reporting-checklists/strobe-cohort/info/#1a) | Indicate the study’s design with a commonly used term in the title or the abstract | 1 |
| Abstract | [#1b](https://www.goodreports.org/reporting-checklists/strobe-cohort/info/#1b) | Provide in the abstract an informative and balanced summary of what was done and what was found | 2 |
| **Introduction** |  |  |  |
| Background / rationale | [#2](https://www.goodreports.org/reporting-checklists/strobe-cohort/info/#2) | Explain the scientific background and rationale for the investigation being reported | 4,5 |
| Objectives | [#3](https://www.goodreports.org/reporting-checklists/strobe-cohort/info/#3) | State specific objectives, including any prespecified hypotheses | 5 |
| **Methods** |  |  |  |
| Study design | [#4](https://www.goodreports.org/reporting-checklists/strobe-cohort/info/#4) | Present key elements of study design early in the paper | 5 |
| Setting | [#5](https://www.goodreports.org/reporting-checklists/strobe-cohort/info/#5) | Describe the setting, locations, and relevant dates, including periods of recruitment, exposure, follow-up, and data collection | 6,7 |
| Eligibility criteria | [#6a](https://www.goodreports.org/reporting-checklists/strobe-cohort/info/#6a) | Give the eligibility criteria, and the sources and methods of selection of participants. Describe methods of follow-up. | 6,7 |
| Eligibility criteria | [#6b](https://www.goodreports.org/reporting-checklists/strobe-cohort/info/#6b) | For matched studies, give matching criteria and number of exposed and unexposed | n/a  Due to the real-world nature of our cohort, we do not have matched cohorts. However, we corrected for confounders using the appropriate tools. |
| Variables | [#7](https://www.goodreports.org/reporting-checklists/strobe-cohort/info/#7) | Clearly define all outcomes, exposures, predictors, potential confounders, and effect modifiers. Give diagnostic criteria, if applicable | 6,7,8 |
| Data sources / measurement | [#8](https://www.goodreports.org/reporting-checklists/strobe-cohort/info/#8) | For each variable of interest give sources of data and details of methods of assessment (measurement). Describe comparability of assessment methods if there is more than one group. Give information separately for for exposed and unexposed groups if applicable. | 6,7,8 |
| Bias | [#9](https://www.goodreports.org/reporting-checklists/strobe-cohort/info/#9) | Describe any efforts to address potential sources of bias | n/a  Our study focused on real-world data, where variability in MRI protocols and patient demographics reflect clinical practice. |
| Study size | [#10](https://www.goodreports.org/reporting-checklists/strobe-cohort/info/#10) | Explain how the study size was arrived at | 6,7 |
| Quantitative variables | [#11](https://www.goodreports.org/reporting-checklists/strobe-cohort/info/#11) | Explain how quantitative variables were handled in the analyses. If applicable, describe which groupings were chosen, and why | 8 |
| Statistical methods | [#12a](https://www.goodreports.org/reporting-checklists/strobe-cohort/info/#12a) | Describe all statistical methods, including those used to control for confounding | 8 |
| Statistical methods | [#12b](https://www.goodreports.org/reporting-checklists/strobe-cohort/info/#12b) | Describe any methods used to examine subgroups and interactions | 8,10 |
| Statistical methods | [#12c](https://www.goodreports.org/reporting-checklists/strobe-cohort/info/#12c) | Explain how missing data were addressed | 8 |
| Statistical methods | [#12d](https://www.goodreports.org/reporting-checklists/strobe-cohort/info/#12d) | If applicable, explain how loss to follow-up was addressed | 8 |
| Statistical methods | [#12e](https://www.goodreports.org/reporting-checklists/strobe-cohort/info/#12e) | Describe any sensitivity analyses | n/a  Sensitivity analyses were not conducted, as our study focused on the feasibility of analyzing real-world, non-standardized MRI data. |
| **Results** |  |  |  |
| Participants | [#13a](https://www.goodreports.org/reporting-checklists/strobe-cohort/info/#13a) | Report numbers of individuals at each stage of study—eg numbers potentially eligible, examined for eligibility, confirmed eligible, included in the study, completing follow-up, and analysed. Give information separately for for exposed and unexposed groups if applicable. | 9,11,12, 13,14 |
| Participants | [#13b](https://www.goodreports.org/reporting-checklists/strobe-cohort/info/#13b) | Give reasons for non-participation at each stage | 9,11 |
| Participants | [#13c](https://www.goodreports.org/reporting-checklists/strobe-cohort/info/#13c) | Consider use of a flow diagram | 9 |
| Descriptive data | [#14a](https://www.goodreports.org/reporting-checklists/strobe-cohort/info/#14a) | Give characteristics of study participants (eg demographic, clinical, social) and information on exposures and potential confounders. Give information separately for exposed and unexposed groups if applicable. | 9,13,14 |
| Descriptive data | [#14b](https://www.goodreports.org/reporting-checklists/strobe-cohort/info/#14b) | Indicate number of participants with missing data for each variable of interest | n/a  We have no overview per variable of interest since participants with missing data of at least one of the variables of interest were excluded |
| Descriptive data | [#14c](https://www.goodreports.org/reporting-checklists/strobe-cohort/info/#14c) | Summarise follow-up time (eg, average and total amount) | 9,14 |
| Outcome data | [#15](https://www.goodreports.org/reporting-checklists/strobe-cohort/info/#15) | Report numbers of outcome events or summary measures over time. Give information separately for exposed and unexposed groups if applicable. | 9,14 |
| Main results | [#16a](https://www.goodreports.org/reporting-checklists/strobe-cohort/info/#16a) | Give unadjusted estimates and, if applicable, confounder-adjusted estimates and their precision (eg, 95% confidence interval). Make clear which confounders were adjusted for and why they were included | 10,12, 13 |
| Main results | [#16b](https://www.goodreports.org/reporting-checklists/strobe-cohort/info/#16b) | Report category boundaries when continuous variables were categorized | 11,13,14 |
| Main results | [#16c](https://www.goodreports.org/reporting-checklists/strobe-cohort/info/#16c) | If relevant, consider translating estimates of relative risk into absolute risk for a meaningful time period | n/a  Not relevant for the purpose of our study |
| Other analyses | [#17](https://www.goodreports.org/reporting-checklists/strobe-cohort/info/#17) | Report other analyses done—eg analyses of subgroups and interactions, and sensitivity analyses | 10,14 |
| **Discussion** |  |  |  |
| Key results | [#18](https://www.goodreports.org/reporting-checklists/strobe-cohort/info/#18) | Summarise key results with reference to study objectives | 15 |
| Limitations | [#19](https://www.goodreports.org/reporting-checklists/strobe-cohort/info/#19) | Discuss limitations of the study, taking into account sources of potential bias or imprecision. Discuss both direction and magnitude of any potential bias. | 17,18 |
| Interpretation | [#20](https://www.goodreports.org/reporting-checklists/strobe-cohort/info/#20) | Give a cautious overall interpretation considering objectives, limitations, multiplicity of analyses, results from similar studies, and other relevant evidence. | 18 |
| Generalisability | [#21](https://www.goodreports.org/reporting-checklists/strobe-cohort/info/#21) | Discuss the generalisability (external validity) of the study results | 15,18 |
| **Other Information** |  |  |  |
| Funding | [#22](https://www.goodreports.org/reporting-checklists/strobe-cohort/info/#22) | Give the source of funding and the role of the funders for the present study and, if applicable, for the original study on which the present article is based | 19 |

The STROBE checklist is distributed under the terms of the Creative Commons Attribution License CC-BY. This checklist was completed using <https://www.goodreports.org/>, a tool made by the [EQUATOR Network](https://www.equator-network.org) in collaboration with [Penelope.ai](https://www.penelope.ai)

**Supplementary Table 3**. Demographics of MS patients who were not on DMT and HC. Continuous data presented in mean + SD or median [range], categorical data presented as count (percentage).

|  | MS w/o DMT | HC | *MS w/o DMT vs HC p-value* |
| --- | --- | --- | --- |
| **Number of subjects** | 20 | 27 |  |
| **Age (years)** | 49 + 8 | 49 + 13 | 0.779 |
| **Sex (F/M)** | 15 / 5 (75%) | 16 / 11 (59%) | 0.355 |
| **Interscan interval (years)** | 4.4 [3.6 – 5.1] | 2.7 [1.8 – 4.1] | < 0.001 |
| **Similarity-index (%)** | 0.21 [0.15 – 0.30] | 0.29 [0.24 – 0.44] | < 0.001 |
| **Annualized PVC WB** | -0.18 + 0.43 | -0.29 + 0.27 | 0.286 |
| **Annualized PVC TGM** | -0.33 + 0.37 | -0.35 + 0.29 | 0.823 |
| **Annualized PVC CGM** | -0.32 + 0.38 | -0.34 + 0.29 | 0.831 |
| **Annualized PVC DGM** | -0.47 [-1.52 – 0.46] | -0.55 [-2.22 – 0.11] | 0.907 |

CGM = Cortical Gray Matter; DGM = Deep Gray Matter; DMT = Disease-modifying treatment; F = Female; HC = Healthy controls; M = Male; MS = Multiple Sclerosis; NA = Not Applicable; PMS = Progressive Multiple Sclerosis; PVC = Percentage Volume Change; RR = Relapsing-Remitting; SD = Standard Deviation; TGM = Total Gray Matter; WB = Whole Brain.

**Supplementary Table 4.** Linear regression models with ΔMSFC_SDMT_ as dependent variable and significant MRI measures as independent variables, with their adjusted outcomes when stepwise adding confounders. Data is presented as estimates of regression coefficient (β) + SE.

| **ΔMSFC_SDMT_** |  |  | **Estimate + SE** | ***p-value*** |
| --- | --- | --- | --- | --- |
|  | **PVC WB** |  | 0.05 + 0.02 | 0.030 |
|  |  | Age | 0.05 + 0.02 | 0.039 |
|  |  | Sex | 0.05 + 0.02 | 0.033 |
|  |  | Cardiovascular risk factors | 0.05 + 0.02 | 0.037 |
|  |  | Education | 0.05 + 0.02 | 0.033 |
|  |  | DMT | 0.05 + 0.02 | 0.038 |
|  |  | MS phenotype | 0.06 + 0.02 | 0.026 |
|  |  | Disease duration | 0.05 + 0.02 | 0.034 |
|  | **PVC TGM** |  | 0.07 + 0.02 | 0.002 |
|  |  | Age | 0.07 + 0.02 | 0.003 |
|  |  | Sex | 0.07 + 0.02 | 0.003 |
|  |  | Cardiovascular risk factors | 0.07 + 0.02 | 0.003 |
|  |  | Education | 0.07 + 0.02 | 0.003 |
|  |  | DMT | 0.07 + 0.02 | 0.004 |
|  |  | MS phenotype | 0.07 + 0.02 | 0.003 |
|  |  | Disease duration | 0.07 + 0.02 | 0.003 |
|  | **PVC CGM** |  | 0.07 + 0.02 | 0.002 |
|  |  | Age | 0.07 + 0.02 | 0.002 |
|  |  | Sex | 0.07 + 0.02 | 0.002 |
|  |  | Cardiovascular risk factors | 0.07 + 0.02 | 0.003 |
|  |  | Education | 0.07 + 0.02 | 0.002 |
|  |  | DMT | 0.07 + 0.02 | 0.003 |
|  |  | MS phenotype | 0.07 + 0.02 | 0.002 |
|  |  | Disease duration | 0.07 + 0.02 | 0.002 |

9HPT = 9-Hole Peg Test; CGM = Cortical Gray Matter; DMT = Disease-modifying treatment; MS = Multiple sclerosis; MSFC_SDMT_ = Multiple Sclerosis Functional Composite Score using SDMT; PVC = Percentage Volume Change; SE = Standard Error; TGM = Total Gray Matter; WB = Whole Brain.

**Supplementary Table 5**. Demographics of MS cohorts used for logistic regression analyses, using a cut-off of -0.16% annualized whole brain volume loss. Data presented in mean + SD or median [range].

|  | Physiological BVL (> -0.16%/year) | Pathological BVL (< -0.16%/year) |
| --- | --- | --- |
| **Number of subjects** | 34 | 28 |
| **Age (years)** | 44 + 8 | 46 + 11 |
| **Sex (F/M)** | 26 / 8 | 24 / 4 |
| **CVD (None / 1 / >2)** | 21 / 9 / 4 | 11 / 12 / 5 |
| **MS-type (RR / PMS)** | 30 / 4 | 23 / 5 |
| **DMT (None / First-line / Second-line)** | 9 / 16 / 9 | 7 / 16 / 5 |
| **Education level (Lower / Higher)** | 15 / 19 | 17 / 11 |
| **Interscan interval (months)** | 52 + 5 | 55 + 4 |
| **Disease duration (months)** | 144 [36 – 396] | 192 [24 – 408] |
| **Baseline EDSS** | 3.3 [1.0 – 6.5] | 3.3 [1.5 – 6.5] |
| **Baseline T25FWT (seconds)** | 5.1 [3.4 – 16.5]***** | 6.0 [3.2 – 15.0]***** |
| **Baseline 9HPT Dominant (seconds)** | 20.9 [15.0 – 38.3] | 20.5 [14.0 – 37.4] |
| **Baseline SDMT** | 53.6 + 13.6 | 49.1 + 13.3 |
| **Baseline MSFC** | 0.4 + 0.7 | 0.3 + 0.7 |

9HPT = 9-Hole Peg Test; CVD = Cardiovascular comorbidities; DMT = Disease-modifying Treatment; EDSS = Expanded Disability Status Scale; F = Female; M = Male; MSFC = Multiple Sclerosis Functional Composite Score; PMS = Progressive Multiple Sclerosis; RR = Relapsing-Remitting; SD = Standard Deviation; SDMT = Symbol Digit Modalities Test; T25FWT = Timed 25-Feet Walk Test. * P < 0.05

**Supplementary Table 6.** Logistic regression models for annualized whole brain volume loss with a cut-off of -0.16% per year as dependent variables and baseline clinical and demographic measures as independent variables. Data is presented as estimates of regression coefficient (β) + SE.

|  | Pathological BVL (< -0.16% per year) | |
| --- | --- | --- |
| EDSS | 0.07 + 0.34 | |
| T25FWT | 0.06 + 0.19 | |
| 9HPT | -0.06 + 0.16 | |
| SDMT | 0.003 + 0.10 | |
| MSFC | -0.42 + 2.89 | |
| Age | 0.04 + 0.04 | |
| Sex (*Male*) | -0.71 + 0.90 | |
| Disease duration | -0.01 + 0.04 | |
| MS type (*RR MS*) | 0.19 + 1.02 | |
| Education level (*Higher*) | -0.34 + 0.64 | |
| Comorbidities (*One \| Two or more*) | 1.12 + 0.75 | 0.65 + 0.85 |
| DMT (*First-line* \| *Second-line*) | 0.54 + 0.78 | -0.12 + 1.01 |

9HPT = 9-Hole Peg Test; BVL = Brain Volume Loss; DMT = Disease-modifying Treatment; EDSS = Expanded Disability Status Scale; F = Female; M = Male; MSFC = Multiple Sclerosis Functional Composite Score; PP = Primary Progressive; RR = Relapsing-Remitting; SDMT = Symbol Digit Modalities Test; SE = Standard Error; T25FWT = Timed 25-Feet Walk Test.
